# Design and Implementation of a Cost-Effective IoT-Based Monitoring and Alerting System for Recirculating Aquaculture Systems (RAS)

**DOI:** 10.3390/s25216692

**Published:** 2025-11-02

**Authors:** Emmanouil E. Malandrakis

**Affiliations:** Laboratory of Applied Hydrobiology, Department of Animal Science, School of Animal Biosciences, Agricultural University of Athens, Iera Odos 75, 11855 Athens, Greece; emalandrak@aua.gr

**Keywords:** IoT, aquaculture, RAS, Raspberry Pi, monitoring, HTTPS, ThingsBoard, sensor networks, alerting system, precision aquaculture

## Abstract

**Highlights:**

**What are the main findings?**

- The developed IoT monitoring system is highly accurate and reliable.
- The system’s integrated alerting function proved critical for operational reliability.

**What are the implications of the main finding?**

- The system enables proactive aquaculture management.
- It provides a validated, scalable blueprint for precision aquaculture.

**Abstract:**

Recirculating Aquaculture Systems (RAS) represent a high-density, controlled-environment fish farming method that requires constant monitoring of critical water quality parameters to ensure high water quality and fish stock health. Manual monitoring is labor-intensive and prone to error, creating a significant risk of catastrophic loss. This work presents the design and implementation of an automated monitoring system built on a Raspberry Pi platform that integrates multiple sensors (temperature, pH, conductivity, water level, and pumps’ functionality) to provide continuous, real-time data acquisition. A key feature is a software-based outlier rejection algorithm that enhances data integrity, and the code is freely available on the GitHub platform for further development. The collected data has been published on the ThingsBoard IoT platform for visualization and historical analysis via the HTTPS protocol. Furthermore, the system implements a proactive alerting mechanism using the Pushover notification service to deliver instant mobile alerts when parameters deviate from predefined thresholds. Commercial solutions cost in the order of thousands of euros, have high maintenance and operational costs, and pose integration and compatibility challenges. This solution provides a reliable, scalable, and cost-effective method for maintaining optimal conditions in a RAS, with hardware costs of less than EUR 150.

## 1. Introduction

Global aquaculture production must increase substantially to meet the protein demands of a growing global population, yet it faces challenges related to environmental sustainability, disease outbreaks, and resource efficiency [1]. Recirculating Aquaculture Systems (RAS) have emerged as a promising solution, enabling high-intensity production with a significantly reduced water footprint by continuously filtering and reusing marine or freshwater [2]. A key technical challenge in RAS management is maintaining a stable and high-quality environment for fish production. Critical water parameters—including temperature, pH, conductivity (a proxy for salinity and total dissolved solids), and dissolved oxygen—must be kept within narrow, species-specific ranges to minimize stress, suppress disease, and optimize growth [3,4]. Furthermore, the mechanical components of the system, such as water pumps and aerators, are vital for life support; their failure can lead to rapid ammonia increase, oxygen depletion, and mass mortalities within a short period of time.

Traditional monitoring protocols rely on manual sampling and visual inspection, which are inefficient, lack the advantages of monitoring through continuous data acquisition, and cannot offer immediate alerts for off-hour emergencies. This delay between a parameter deviation and its detection is a primary source of production risk [5]. The integration of Internet of Things (IoT) technology marks a paradigm shift, enabling automated, real-time monitoring and control, a concept now central to the emerging field of Precision Aquaculture [6,7]. IoT-based systems can provide a continuous stream of data, facilitate remote management, and enable data-driven decision-making, ultimately enhancing productivity, sustainability, and animal welfare [8].

Several studies have demonstrated the feasibility of THE IoT for aquaculture monitoring. For instance, systems have been developed for pond-based aquaculture [9] and for monitoring specific parameters such as dissolved oxygen [10]. However, many existing solutions are fragmented, focusing on a limited set of parameters, or depend on proprietary and expensive hardware/software platforms. In most cases, Arduino is used as the controller board due to its ability to directly connect with analog sensors [11]. Another controller used is the ESP032, which is known for being powerful, versatile, and cost-effective [12].

The current work presents the development of a comprehensive, open-source, integrated, and low-cost RAS monitoring system, priced under EUR 150, that addresses these gaps. The system utilizes a Raspberry Pi, open-source software, and a modular design to enable continuous monitoring of a broad range of water quality and operational parameters. A key contribution is the implementation of a software-based algorithm for outlier rejection to ensure high data fidelity and false alarms. Furthermore, the system integrates with industry-standard IoT platforms for visualization and incorporates a robust push notification alert system, providing a complete solution for modern, risk-free aquaculture operations.

## 2. Materials and Methods

### 2.1. Hardware

**Figure 1:**
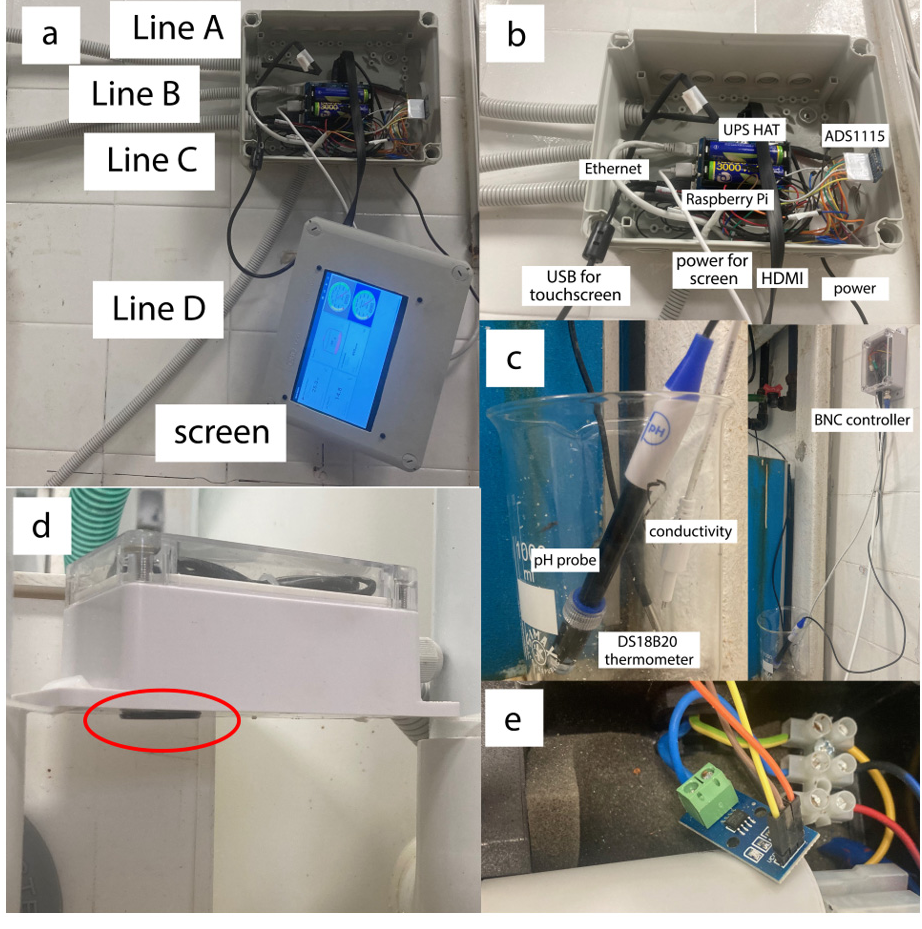
(**a**). The system is mounted on the wall, with a 7-inch screen. Line A connects the ultrasonic sensor, Line B connects the ACS712-20A recirculation pump sensor, Line C connects the conductivity, pH, and temperature sensors, and Line D connects the dispensing pump sensor. (**b**) The housing for the Raspberry Pi 3 B+ single-board computer, stacked directly on top, features a UPS HAT and an ADS1155 analog-to-digital converter for data acquisition. Network connectivity is provided via an Ethernet cable to ensure stable access. The display setup includes a dedicated USB cable for touchscreen functionality and an HDMI cable for video signal transmission. Primary power is supplied through a power supply unit connected to the UPS HAT. (**c**) In the left image, the thermometer, conductivity, and pH sensors are shown. In the right image, the BNC controller is depicted within its waterproof housing. (**d**) The ultrasonic sensor (highlighted in the red ellipse) is installed at the bottom of the waterproof housing. (**e**) The pump line is equipped with an ACS712-20A current sensor.

The system was designed with a modular architecture, consisting of a sensing layer, a processing and communication layer, and a cloud-based visualization and alerting layer. The system was built around a Raspberry Pi 3 Model B+ 1 GB single-board computer (Raspberry Pi Foundation, Cambridge, UK), which acts as the central processing unit. The system integrates a suite of sensors for comprehensive monitoring, beginning with a DS18B20 digital thermometer (Analog Devices, Wilmington, MA, USA) for precise water temperature readings via a 1-Wire interface. A high-resolution 16-bit ADS1115 (Texas Instruments, Dallas, TX, USA) Analog-to-Digital Converter (ADC) was employed to interface with analog sensors, including a BNC pH probe and a conductivity electrode. The ADS1115 ADC, which was used as the front end for signal conversion, uses an internal 2.048 V voltage reference to ensure measurement stability. Filters were not applied in the ACS712-20A current sensor and pH sensor. Cables used in all connections were F/UTP, which have a simple foil shield wrapped around all 4 twisted pairs. GND from analog sensors was connected to the ADC GND. The same ADC also processed data from two analog current sensors (ACS712-20A, Allegro Microsystems, Manchester, NH, USA), which monitored the recirculating and dispensing pumps by measuring their operational currents. An ultrasonic distance sensor (JSN-SR04T, Shenzhen Gaituo Electronic Technology, Guangdong, China) was connected directly to the Raspberry Pi’s GPIO pins to provide non-contact water level measurement in the header tank. The water level was used to calculate the system’s water volume. A UPS HAT for Raspberry Pi (Waveshare, Shenzhen, China) was installed to provide uninterrupted power and protect against data corruption during electrical current interruptions or shortages. The UPS is physically connected by aligning it with the Raspberry Pi’s 40-pin GPIO header. Two lithium-ion batteries (type 18650, 3000 mAh, 3.7 V, Energenie, Gembird, Almere, The Netherlands) are connected to the dedicated connector on the UPS. A 7-inch 1024 x 600 IPS 5-point capacitive touchscreen HDMI Display (Hzwdone, China) was connected to the Raspberry Pi board, serving as the primary UI for real-time data visualization and local system control. Additionally, this system was connected to a router through Ethernet and can be monitored through the VNC protocol from a PC within the local network. Finally, the system was installed in a compact, plug-and-play recirculation filtration system (TMC System 5000 Marine, Tropical Marine Centre, Hertfordshire, UK), which is connected to 12 174 L aquaria. All the system components are presented in Figure 1. A Bill of Materials is presented in Supplementary Table S1.

All sensors underwent a rigorous calibration procedure to ensure measurement traceability and define measurement uncertainty. Calibrations were performed under laboratory conditions at 21.4 °C ± 1 °C. The DS18B20 digital temperature sensors were calibrated against a certified mercury-in-glass thermometer (−30–50 °C, Gilson, Lewis Center, OH, USA) across the operational range of 4 °C to 40 °C. In total, forty-five (45) readings were used for the temperature sensor calibration. The calculated expanded uncertainty (k = 2) for temperature measurement was ±0.096 °C.

The pH probe was calibrated using a three-point method with NIST-traceable buffer solutions at pH 4.01, 7.00, and 10.01. The probe’s readings were simultaneously verified against a calibrated Hach HQ11d pH meter (Hach Europe, Dusseldorf, Germany). Forty-three (43) readings were collected after signal stabilization. The operational range of the pH sensor is 4–10. The conductivity electrode was calibrated against a certified HI2003-EDGE conductivity meter with twenty-nine (29) measurements. The expanded uncertainties (k = 2) were determined to be ±0.058 for pH and ±0.60µS/cm for conductivity.

For water level measurement, the ultrasonic distance sensor was calibrated against a certified folding ruler (EU accuracy class III). The water level in the container during calibration varied from 1833 L and 2043 L, and at each of the 29 points, sensor readings were recorded and compared to the direct ruler measurement. For the specific setup, the working range of the sensor is 0–2340 L, which is the maximum capacity of the system. The sensor itself has a 25 cm to 400 cm working range. The core software is a single Python script (Python version 3.11.2) structured around a SensorMonitor class, promoting maintainability. The data acquisition module implements methods for reading individual sensors. A key code part within this module is the ‘get_filtered_sample’ method. This function is designed to acquire multiple samples (with a configurable default of 10) and subsequently sort them. A number of the highest and lowest outliers, with a default of two, are then discarded.

The function returns the mean of the remaining six values, implementing a filtering technique that drastically reduces noise and outliers to ensure high data integrity. A communication module utilizes an HTTPS client library to establish secure connections to the ThingsBoard IoT platform. HTTPS client and device credentials are employed for each sensor metric. HTTPS client was configured with SSL/TLS encryption using the system’s Certificate Authority bundle. This specific architectural decision provides extreme flexibility for building dashboards and configuring independent alert rules on the ThingsBoard server for each distinct data stream. The data management module is responsible for two primary functions. Firstly, it performs local logging by appending all sensor data with a timestamp to a CSV file on the Raspberry Pi’s SD card; a copy is simultaneously written to a USB drive to ensure data backup. Secondly, the module handles cloud publishing by transmitting the filtered sensor readings to their respective HTTPS topics on the ThingsBoard platform. Finally, an alerting module performs a critical monitoring function through its ‘check_alerts’ method. This method compares the latest sensor readings against a dictionary of thresholds (e.g., temp_low: 19.0, ph_high: 8.5, etc). Upon detection of any threshold exceeding, the system composes a message and executes the ‘send_pushover_notification’ method. This method utilizes the Pushover API to dispatch the message, resulting in an immediate push notification to the designated mobile device (smartphone and/or tablet).

### 2.2. Operational Workflow and Error Handling

The system operates on a timed loop controlled by a Timer object from the threading module. Upon execution, the SensorMonitor class initializes all hardware and network connections. The main loop, *collect_and_publish*, is then initiated and repeats at a configurable interval, with a programmed period (e.g., ten minutes). Each operational cycle consists of sequentially reading and filtering data from all attached sensors, publishing the resultant data to the ThingsBoard cloud platform, and logging the data with a timestamp to a local CSV file. Subsequently, the system checks the newly acquired values against a set of predefined alert thresholds and sends push notifications via the Pushover API if any breaches are detected. Finally, the loop schedules its next cycle before terminating. Safe settings (thresholds, intervals, hardware pins) and secret tokens (API keys, passwords, device tokens) are not hardcoded and are referenced to the configuration file and environment variables file, respectively. System parameters can be adjusted through environment variables and YAML configuration files, without altering the core source code.

The system architecture is designed for continuous 24/7 operation, incorporating several key reliability features. Extensive try-except blocks encapsulate every critical operation, including individual sensor reads, network calls, and file operations. This design prevents a single point of failure, such as a disconnected sensor or a network outage, from causing a complete application crash. Furthermore, comprehensive logging is implemented using the Python logging module, which is configured to record informational messages, warnings, and errors to both the console and a permanent log file. This feature is useful for remote debugging and system status monitoring. To ensure robustness, the system also implements a safe shutdown procedure. A two-stage battery management protocol ensures system integrity. When the battery level drops to 30%, a warning message is sent through Pushover. If no action is taken and the battery level further depletes to 10%, the system automatically initiates a safe shutdown sequence to prevent data loss or hardware damage. A dedicated cleanup method ensures that upon a manual interrupt or a fatal error, all hardware interfaces, such as GPIO, are reset to a safe state, and all network connections are properly closed before application termination.

### 2.3. Experimental Implementation

The experiment was conducted for one month in the RAS equipped with the IoT system. One hundred twenty *Sparus aurata* juveniles (85.6 ± 8.5 g) were acquired from a commercial farm and randomly allocated into the 12 tanks. The fish were maintained under a 12 h:12 h light/dark photoperiod and hand-fed to satiation twice daily. The system chiller was set to 21 °C, and the pH was regulated with NaOH when it fell below 7. The dispersion pump was set to 1900 rpm using an inverter, and the recirculation pump was operating at its default current (approx. 2800 RPM). The facility is licensed to conduct experimental protocols under license number EL 25 BIO exp 046.

Statistical analysis evaluated system performance against reference standards. Agreement for environmental and volume sensors was quantified using Mean Absolute Error (MAE) and Root Mean Square Error (RMSE), while linear correlation was assessed with the coefficient of determination (R^2^). Method comparison was further validated through Bland–Altman analysis, Passing–Bablok regression, and the non-parametric Kendall’s τ rank correlation coefficient. Pump performance was reported as the mean speed ± standard deviation.

## 3. Results

**Figure 2:**
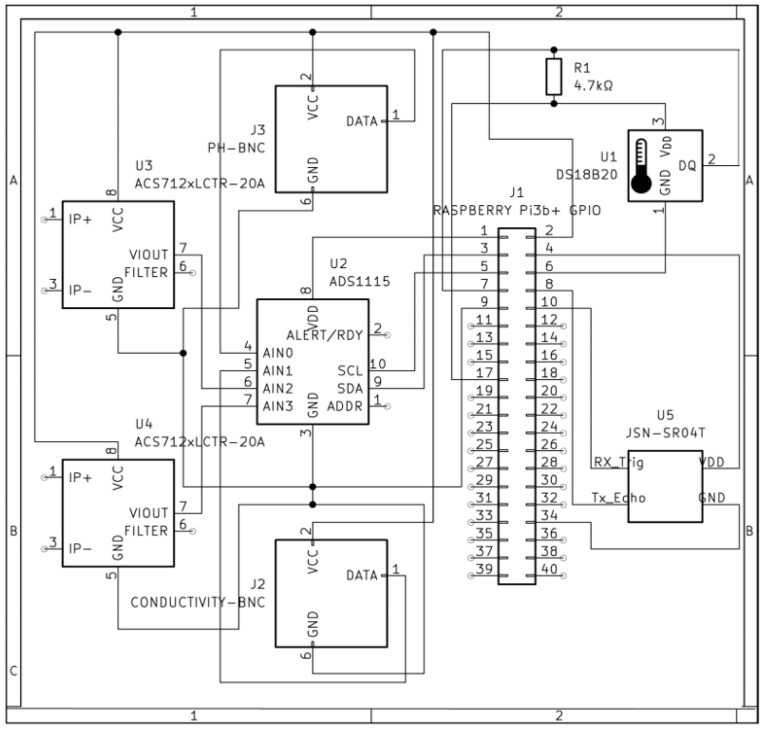
Electrical diagram of the RAS monitoring system.

**Figure 3:**
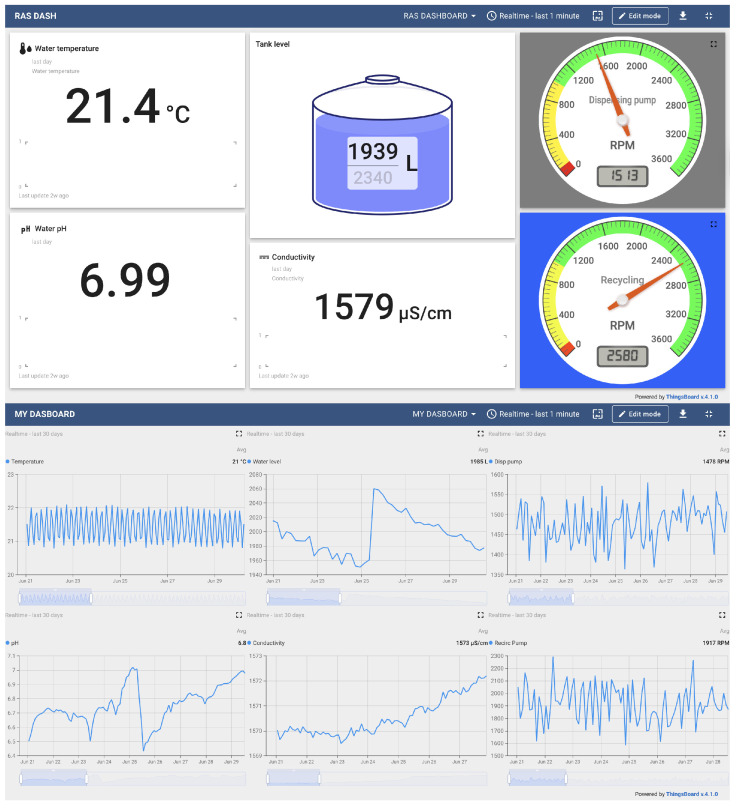
Two different dashboards that were developed in the ThingsBoard platform to monitor sensor data. The upper dashboard presents current data in a 10 min interval, and the lower presents a time series according to the settings.

The electrical diagram of the monitoring system is presented in Figure 2. A system flowchart is presented in Supplementary Figure S1. The developed RAS monitoring system successfully acquired, processed, and transmitted data from all integrated sensors (temperature, pH, conductivity, water volume, and pumps’ function) over a continuous monthly validation period. The system’s performance was evaluated during a power outage, maintaining operation for 2.5 h. The system executed its main loop at the predefined 10 min interval without failure, resulting in a total of 4302 data collection cycles. The statistics of the data received in the log file are presented in Table 1. The two different dashboards that have been developed in ThingsBoard are presented in Figure 3.

**Figure 4:**
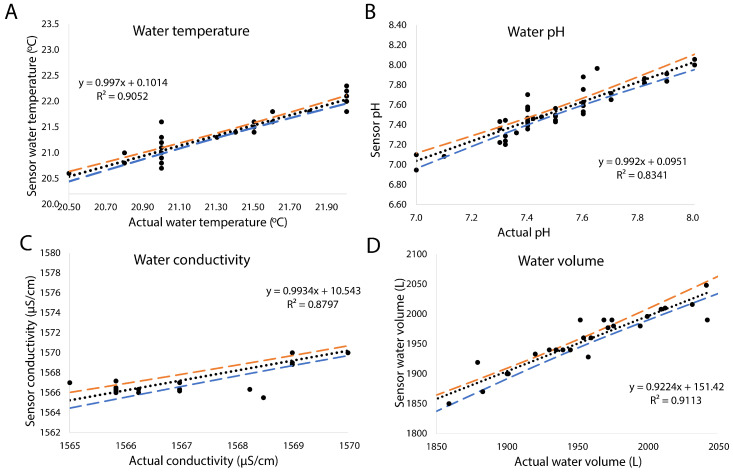
Calibration curves for temperature ((**A**); *n* = 45), pH ((**B**); *n* = 43), conductivity ((**C**); *n* = 36), and water volume ((**D**); *n* = 29) sensors (spotted lines). Red dashed lines represent the upper 95% confidence intervals, and blue dashed lines represent the lower 95% confidence intervals.

The performance of the sensor system was validated against standardized sensors. For environmental parameters, the sensor means were in close agreement with the reference means: Temperature (21.43 ± 0.49 vs. 21.36 ± 0.46 °C), pH (7.46 ± 0.21 vs. 7.50 ± 0.23), and conductivity (1570.3 ± 3.2 vs. 1569.8 ± 3.8 µS/cm). This was supported by low error metrics, with Mean Absolute Error (MAE) values of 0.12 °C, 0.08, and 0.84 µS/cm, respectively. The strong correlation (Figure 4) coefficients (R^2^: 0.91, 0.83, 0.88, and 0.91) confirm a high linear correlation between the sensor and reference data. For water volume measurement, the system demonstrated a strong correlation (R^2^ = 0.91) with a reference standard, characterized by a low absolute error (MAE = 11.7 L, RMSE = 17.4 L). The dispensing pump control systems performed as intended with a mean speed of 1897 and a low standard deviation (217 RPM). The results from the Bland-Altman analysis are presented in Supplementary Figure S2 and Supplementary Table S2. The central solid yellow line represents the mean difference (bias) between the two measurement methods, while the upper and lower dashed lines indicate the 95% limits of agreement (mean difference ± 1.96 standard deviations of the differences. The results were further supported by Passing-Bablok regression (Supplementary Figure S3). Kendall’s τ, which is a non-parametric measure of the ordinal association between sensors and calibrators’ data, varied between 0.77–0.85 (Supplementary Table S2). During the testing period, the recirculation pump sensor provided high variation for the current measurements and alarmed when the pump stopped due to overheating.

## 4. Discussion

In aquaculture, significant research and development efforts have been dedicated to devising automated systems for comprehensive monitoring and timely alerting [13,14,15,16,17,18,19,20] when water parameters deviate from normal values. The system presented in this work provides a unified platform for monitoring critical RAS parameters such as temperature, pH, conductivity, water level, and pumps’ function. The use of the ADS1115 Analog-to-Digital Converter and the software filtering algorithm ensures stable and reliable sensor readings, free from the electrical noise commonly found in Raspberry Pi-based projects. The use of individual HTTPS device tokens for each data stream, while creating more initial setup on the IoT platform, contributes to dashboard and alert rule flexibility. The integration of mobile push messaging offers a highly dependable, low-latency alerting mechanism, outperforming traditional email-based notifications.

An Arduino-based system has been developed for Asian seabass fish farming, exhibiting similar accuracy levels [15], which incorporates important sensors (e.g., dissolved oxygen, ammonia). These sensors are effective and important, but their use may increase the cost. Alternatively, a current sensor (ACS712) can monitor the air generator’s functionality in place of a DO sensor, though it should be noted that generator failure is not the sole cause of oxygen depletion in fish tanks.

In general, the use of high-quality sensors is strongly recommended to ensure data integrity. A future enhancement would be to make these parameters user-configurable via a web interface file that is read and configured at runtime, eliminating the need to modify the code for adjustments. Although machine learning and AI have been developed, the generation of data will enhance the ability to predict water parameters [21], but so far, auto-calibration of the sensors is not possible.

This work presents a low-cost and robust IoT solution for monitoring RAS [22], aligning with the global trend towards Precision Aquaculture. The system automates the tedious and error-prone task of manual data collection, provides real-time visibility into system health via a cloud dashboard, and offers immediate notification of critical issues, dramatically reducing the risk of stock loss. Furthermore, the observed low mean difference indicates minimal systematic error, suggesting no consistent over- or under-estimation by the sensors. Indeed, the alarm for pump failure via push messaging was crucial in preventing mortalities among the fish during the experimental period. Furthermore, the ThingsBoard platform can be installed on mobile devices, and the user can review data in real-time [23].

The developed system serves as a base for future expansion in several key areas. The first step involves the addition of new sensors. Parameters such as dissolved oxygen, oxidation–reduction potential (ORP), ammonia, and ozone gas are critical for advanced RAS management and can be integrated to provide a more comprehensive monitoring solution. The next area focuses on actuation and control to establish a closed-loop system. This stage involves implementing sensors to continuously monitor the system’s output, feeding this real-time data back to the system, which then compares this feedback to the desired target and calculates any necessary corrective commands. These commands are sent to actuators, which physically adjust the system to minimize the error and achieve precise, autonomous regulation. This would integrate capabilities for automatically activating pumps, heaters, chillers, or valves for dissolved oxygen or pH correction based on the real-time sensor readings, thereby transitioning the platform from passive monitoring to full automation [24]. Furthermore, advanced analytics and predictive maintenance represent a significant opportunity [25]. Integrating machine learning models on the cloud platform could enable the forecasting of component failures, such as pumps or mechanical filters, based on trends in current data. These models could also optimize feeding schedules based on historical water quality trends [26] and taking into account fish behavioral data [27].

Future iterations of the system could greatly enhance its functionality by integrating flow meters and energy monitors, elevating it from a basic operational tool to a comprehensive resource management platform [28]. The addition of flow meters would allow for the precise quantification of both total system water usage and recirculation rates, providing critical data to minimize water consumption and verify hydraulic retention times for the biofilter. Concurrently, energy monitors attached to major components like pumps, chillers, and aerators would provide detailed tracking of energy consumption across the entire operation [29]. By correlating this energy data with environmental parameters and stocking densities, operators could identify inefficiencies, optimize equipment schedules, and calculate key metrics such as Feed Conversion Ratio (FCR) or biomass gained per unit of energy consumed [30]. This integrated approach to monitoring both water and energy flows is fundamental for improving the sustainability and economic viability of aquaculture operations. Finally, future iterations could integrate flow meters and energy monitors to provide a holistic view of resource use efficiency [31]. Monitoring energy and water consumption is a key metric for improving the sustainability and economic viability of aquaculture operations [32].

This system demonstrates the powerful application of consumer-grade IoT technology to solve problems in aquaculture, enhancing both productivity and sustainability. By providing a blueprint for a scalable, open-source monitoring solution, it contributes to the democratization of precision technologies for aquaculture facilities.

## 5. Conclusions

This study successfully designed, implemented, and validated a comprehensive, low-cost, and open-source IoT monitoring system for Recirculating Aquaculture Systems (RAS). The system, built around a Raspberry Pi and open-source software, demonstrated robust and reliable 24/7 operation over a one-month experimental period, autonomously collecting and processing over 4300 data cycles. The key highlights of this work are threefold. First, the system integrates an array of critical parameters—water temperature, pH, conductivity, water volume, and pump operational status—into a single, unified platform. The implementation of a software-based outlier rejection algorithm ensured high data fidelity, which was confirmed through validation against reference sensors, showing strong correlation (R^2^ values from 0.83 to 0.91) and low error metrics. Second, the system architecture effectively bridges the gap between data acquisition and actionable insights. By leveraging the ThingsBoard IoT platform for cloud-based visualization and the Pushover API for instant push notifications, the system provides real-time operational visibility and immediate alerts for critical parameter deviations. This capability was proven crucial during the experiment, when a pump failure alert prevented potential mass mortality. Finally, the project fulfills its goal of providing a low-cost (under EUR 150), open-source solution that addresses the limitations of existing proprietary systems. Its modular design ensures scalability and ease of expansion for future enhancements.

In conclusion, this work provides a validated blueprint for the democratization of precision aquaculture technologies. By automating manual monitoring tasks and enabling data-driven decision-making, the system significantly reduces production risks, enhances operational efficiency, and contributes to the sustainability and economic viability of modern aquaculture operations. Future development will prioritize the integration of actuation for closed-loop control, the addition of advanced sensors, and the implementation of predictive analytics using machine learning—further enhancing the capabilities of intelligent aquaculture management.

## Figures and Tables

**Table 1:** Parameters measured by the installed reference sensors (mean ± SD), mean absolute error (MAE), root mean square error (RMSE), and R-squared.

Parameter	Sensor Mean	Reference Mean	Expanded Uncertainty (U) (k = 2)	Mean Absolute Error (MAE)	Root Mean Square Error (RMSE)	R^2^
Temperature	21.43 ± 0.49 °C	21.36 ± 0.46 °C	0.096 °C	0.12 °C	0.15 °C	0.91
pH	7.46 ± 0.21	7.50 ± 0.23	0.058	0.08	0.11	0.83
Conductivity	1570.3 ± 3.2 µS/cm	1569.8 ± 3.8 µS/cm	0.60 µS/cm	0.84 µS/cm	1.44 µS/cm	0.88
Water volume	1970 ± 77 L	1947 ± 56 L	11.54 L	11.7 L	17.4 L	0.91
Recirculation pump	2800 ± 783 RPM	2800 RPM (set)				
Dispensing pump	1897 ± 217 RPM	1900 RPM (set)				

## Data Availability

The Python code (Python version 3.11.2) supporting sensor function is available on GitHub: https://github.com/emalandrak28/RAS-monitoring (accessed on 29 October 2025). The open-source IoT platform, ThingsBoard, can be found at the following link: https://thingsboard.io/ (accessed on 29 October 2025).

## Supplementary Materials

The following supporting information can be downloaded at: https://www.mdpi.com/article/10.3390/s25216692/s1, Table S1: Bill of Materials (BOM) with prices and suppliers. All values were rounded to the closest integer; Figure S1: System flowchart; Figure S2: Bland-Altman plots of temperature (A), pH (B), conductivity (C), and water level (D). The yellow solid line represents the mean difference (bias), while the dashed green lines represent the lower 95% and upper 95% limit of agreement; Figure S3: Passing-Bablok regression lines for temperature (A), pH (B), conductivity (C), and water level (D). The dotted line is the x = y line, and the red line is the Passing-Bablok regression line; Table S2: Bland-Altman statistics and Kendall’s τ for temperature (°C), pH, conductivity (μS/cm) and water volume (L) sensors.

## Abbreviations

The following abbreviations are used in this manuscript:

ADC	Analog-to-Digital Converter
API	Application Programming Interface
CSV	Comma-Separated Value
DO	Dissolved Oxygen
GPIO	General Purpose Input/Output
GUI	Graphical User Interface
IoT	Internet of Things
MAE	Mean Absolute Error
HTTPS	Hypertext Transfer Protocol Secure
ORP	Oxidation–Reduction Potential
RAS	Recirculating Aquaculture System
RMSE	Root Mean Square Error
RPM	Revolutions Per Minute
UI	User Interface
UPS HAT	Uninterruptible Power Supply Hardware Attached on Top
USB	Universal Serial Bus
VNC	Virtual Network Computing
