# Supplementary material for: Design and Implementation of a Cost-Effective IoT-Based Monitoring and Alerting System for Recirculating Aquaculture Systems (RAS)
Source: Sensors (Basel). 2025 Nov 2;25(21):6692. doi: 10.3390/s25216692 (PMC12609693; doi:10.3390/s25216692)
Supplement: Supplementary file 1 [file sensors-25-06692-s001.zip › sensors-3891009-supplementary.pdf]

Supplementary Table S1 Bill of Materials (BOM) with prices and suppliers. All values were rounded to the closest integer.

Description	Supplier	Quantity	Unit cost	Cost
**Raspberry Pi 3 Model B+**	www.nettop.gr	1	45	45
**Waveshare UPS HAT for Raspberry Pi (2 x 18650)**	www.hellasdigital.gr	1	20	25
**Housing OBO BETTERMANN 240X190mm IP66**	www.meidanis.gr	1	10	10
**7 Inch Monitor 1024x600 IPS 5-Points Capacitive Touchscreen Drive**	www.aliexpress.com	1	21	21
**ADS1115 I2C 16-Bit ADC 4 channel module**	www.nettop.gr	1	6	6
**Hall current sensor module ACS712-20A**	www.aliexpress.com	2	3	6
**Sonoff IP66 waterproof case**	www.hellasdigital.gr	2	4	8
**DS18B20 temperature sensor**	www.aliexpress.com	1	2	2
**Water conductivity sensor**	www.aliexpress.com	1	4	4
**BNC pH electrode probe**	www.ebay.com	1	15	15
**JSN-SR04T ultrasonic sensor**	www.aliexpress.com	1	5	5
**Total**				**147**

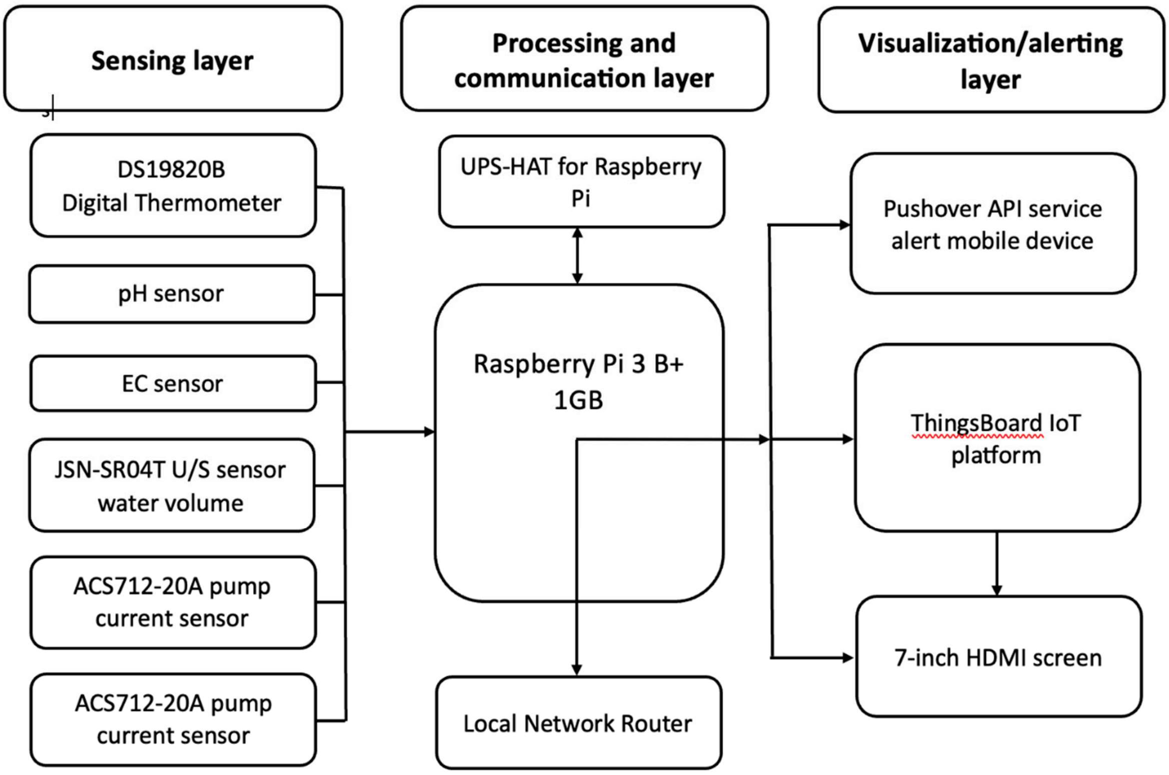

Supplementary Figure S1 System flowchart

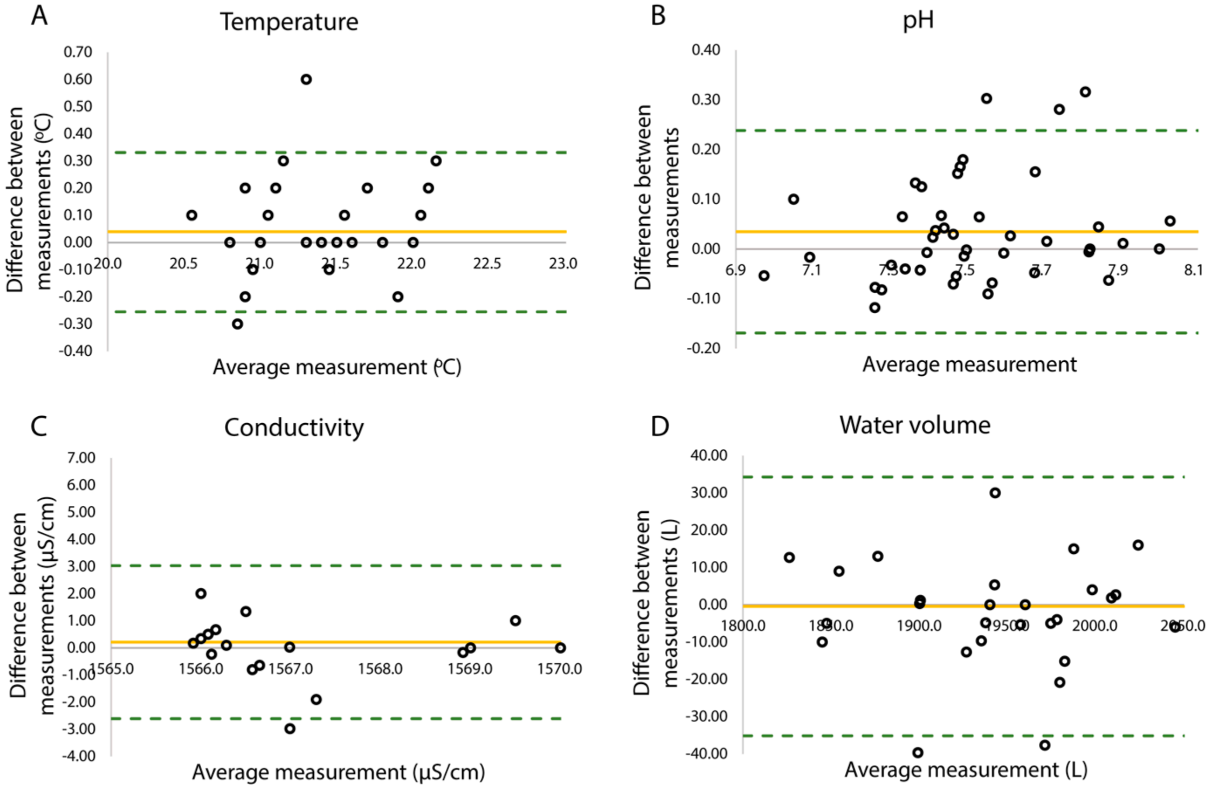

Supplementary Figure S2 Bland-Altman plots of temperature (A), pH (B), conductivity (C), and water level (D). The yellow solid line represents the mean difference (bias), while the dashed green lines represent the lower 95% and upper 95% limit of agreement.

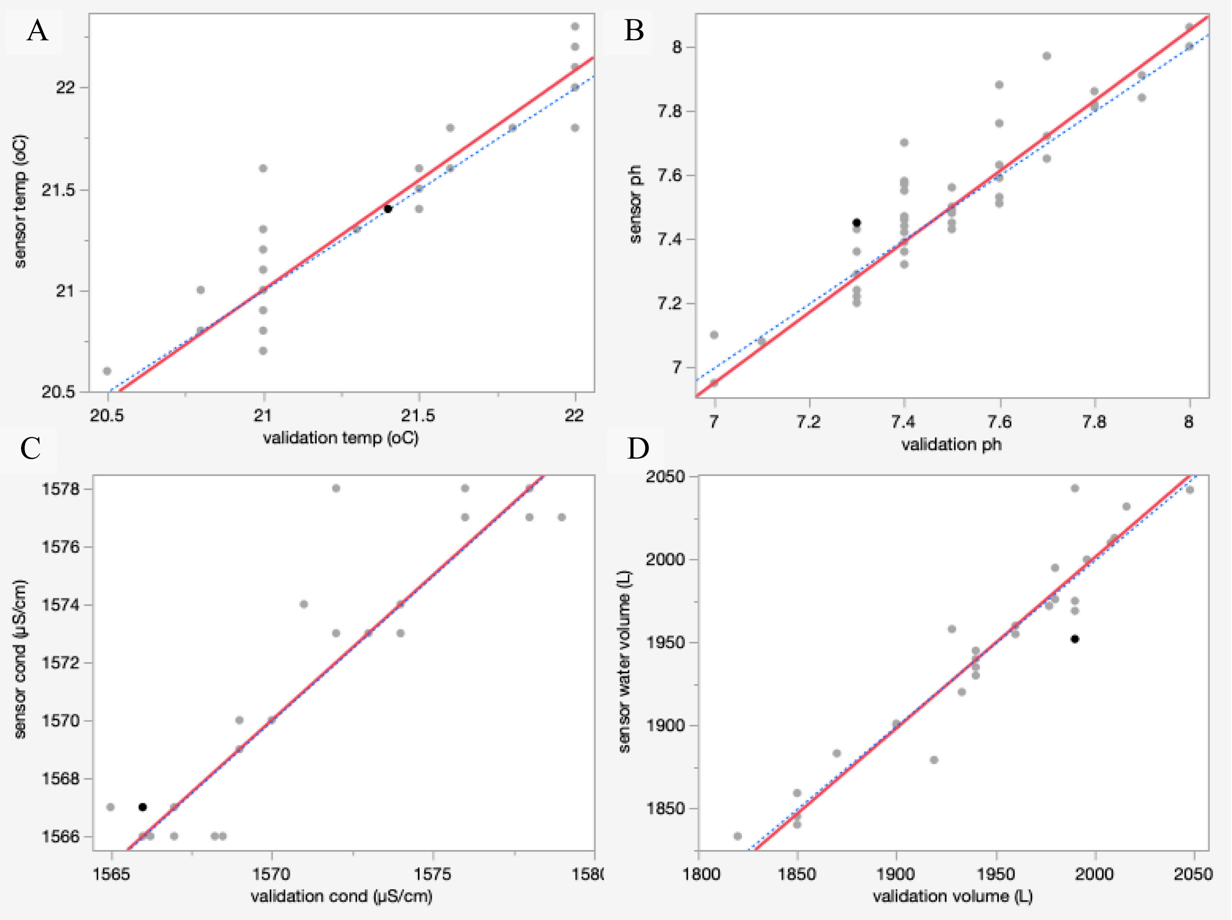

Supplementary Figure S3 Passing-Bablok regression lines for temperature (A), pH (B), conductivity (C), and water level (D). The dotted line is the x=y line, and the red line is the Passing-Bablok regression line.

Supplementary Table S2 Bland-Altman statistics and Kendall's τ for temperature (°C), pH, conductivity (µS/cm) and water volume (L) sensors

	Temperature (°C)	pH	Conductivity (µS/cm)	Water volume (L)
**Bias (Mean Difference)**	0.04	0.02	0.21	-0.42
**Standard Deviation**	0.15	0.10	1.44	17.71
**Upper LoA**	0.33	0.24	3.03	34.29
**Lower LoA**	-0.26	-0.17	-2.61	-35.13
**Kendall's τ**	0.84	0.78	0.77	0.85
